# Successful treatment of osteosarcoma in a pregnant woman with survival of the gestational product: A case report and literature review

**DOI:** 10.3892/mi.2024.197

**Published:** 2024-10-09

**Authors:** Carlos Abraham Orpinel-González, Marcos Iglesias-González, Joel Herrera-Loya, Carlos Arturo Martínez-Méndez, Aaron Alberto Ramírez-Torres, Raúl Gerardo Ramírez-Medina

**Affiliations:** 1 Department of Traumatology and Orthopedics, Hospital Star Médica Chihuahua, Chihuahua 31110, Mexico; 2 School of Medicine, Autonomous University of Chihuahua, Chihuahua 31000, Mexico; 3 Department of General Surgery, Christus Muguerza Chihuahua Park Clinic, Chihuahua 31000, Mexico; 4 CECan State Cancer Center, Chihuahua 31000, Mexico

**Keywords:** osteosarcoma, pregnancy, adjuvant chemotherapy, gestational product survival, surgical resection

## Abstract

Osteosarcoma (OS) is the most prevalent bone neoplasm of mesenchymal origin, accounting for 20% of all bone tumors worldwide. It mainly affects the marrow of long bones, and its diagnosis is more common among adolescents and the geriatric population. Histologically, it is characterized by high cellular variability, abundant osteoid and fibrotic material. In the early stages, it presents with only local symptoms such as pain, edema and limited joint mobility. This neoplasm, when detected promptly, is associated with a favorable prognosis and can be effectively treated through surgical removal and adjuvant therapy. The development of tumors in pregnant women is rare, and the occurrence of osteosarcoma is even more exceptional, with only 10 cases documented in the literature. Given its rarity, the present study describes the case of a female patient with OS diagnosed in the first trimester of pregnancy, where the patient responded well to treatment, resulting in no adverse effects on the pregnancy outcome.

## Introduction

Osteosarcoma (OS) is a highly prevalent malignant tumor of mesenchymal origin, accounting for 20% of all bone tumors (1). It occurs mainly in adolescents and the geriatric population (2). Etiologically, it is linked to genetic mutations on chromosome 6, previous exposure to radiotherapy, a high birth weight, repeated bone trauma and the African race (3). Mortality rates vary according to the histological grade, with ~3.1 deaths per million affected individuals annually (4). OS typically manifests in the long bones of the appendicular skeleton (5), primarily as localized lesions in the bone marrow; however, it can also occur in the periosteum, bone cortex and surrounding soft tissues (6). Histologically, it contains variable amounts of osteoid and fibrotic material, exhibiting different cellular predominance of osteoblasts, chondroblasts, or fibroblasts. It also exhibits various degrees of cellular degeneration and varying mitotic rates (7). Clinically, it presents with pain, swelling, limited joint mobility, and, in some cases, pathological fractures (8). Surgery combined with adjuvant chemotherapy is regarded as the optimal treatment for this neoplasm (9). Prompt intervention is crucial, as the tumor can metastasize to the lungs, requiring timely treatment to minimize mortality (10). Regrettably, diagnosis can be delayed in some patients as the symptoms may be mistaken for more common conditions, as observed when OS occurs during pregnancy. Currently, the treatment of OS during pregnancy may be controversial due to potential risks to the fetus (5).

Therefore, the present study describes the case of a pregnant woman diagnosed with OS in the appendicular skeleton during the first trimester. She underwent surgical treatment and adjuvant chemotherapy, exhibiting a positive response to treatment and successful survival of the gestational product.

## Case report

The medical care for the patient discussed in the present case report was provided at Star Médica Hospital Chihuahua, Chihuahua, Mexico. The patient's care was administered from August, 2022 to January, 2023, in accordance with the standard protocols established by the institution and the specific pathology presented. As of the drafting of this clinical case report, both the mother and the infant are in good overall health.

**Figure 1:**
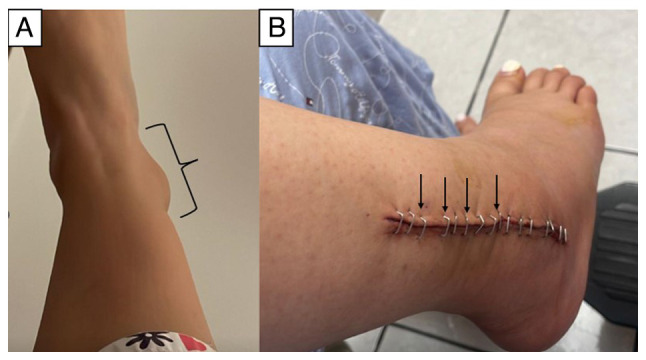
Original images from the tumor site. (A) Original image of the tumor site provided by the patient before commencing the diagnostic approach. (B) Image of the tumor site obtained 10 days after the surgical treatment. Adequate coping with the surgical wound can be observed.

**Figure 2:**
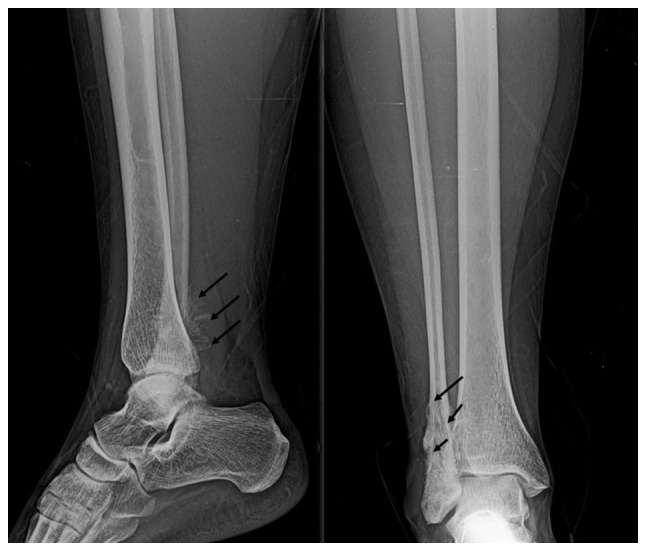
Anteroposterior and lateral ankle radiography: Shown are hypodense lesions located in the distal diaphysis of the right fibula with discontinuity of the cortex with the radiological sign of ‘rising sun’ (black arrows).

**Figure 3:**
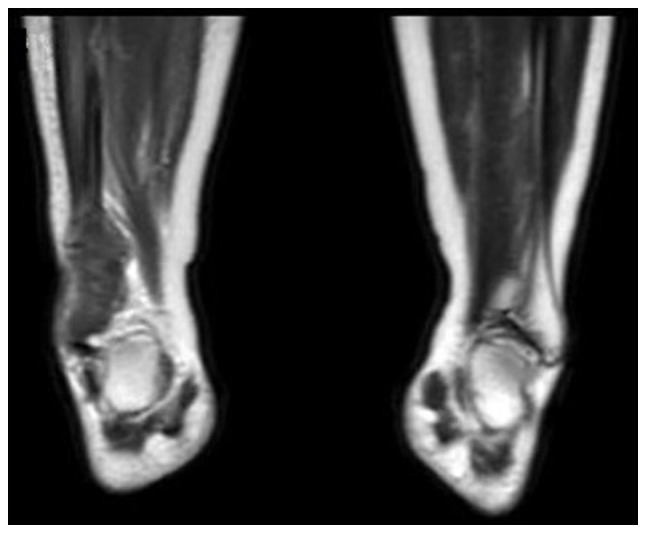
Magnetic resonance imaging of the ankle coronal section: Intense hypo tissue is observed in the distal portion of the right fibula, evidencing extension to soft tissues.

A 21-year-old female with no notable medical history was in the first trimester of a physiological pregnancy, managed with mineral supplements and multivitamins. Around the 11th week of gestation, the patient reported swelling and localized pain in the distal third of her right tibia and fibula region (Fig. 1A). Anterior-posterior and lateral X-rays of the right ankle revealed granular lesions of variable radiographic density in the region of the right lateral malleolus (Fig. 2). Given the suspicion of malignancy, contrast-enhanced magnetic resonance imaging (MRI) of the abdominal-pelvic region, chest, and right ankle was conducted. The MRI revealed increased volume and signal intensity in the soft tissues surrounding the lateral malleolus. A non-mineralized bony matrix lesion was identified in the distal diaphysis of the fibula, appearing hypointense on the T1 sequence and hyperintense in the proximal diaphysis, with irregular margins measuring 66x30x20 mm. The lesion caused cortical destruction and extension into adjacent soft tissues. Post-contrast imaging revealed avid and heterogeneous enhancement of the affected region (Fig. 3).

**Figure 4:**
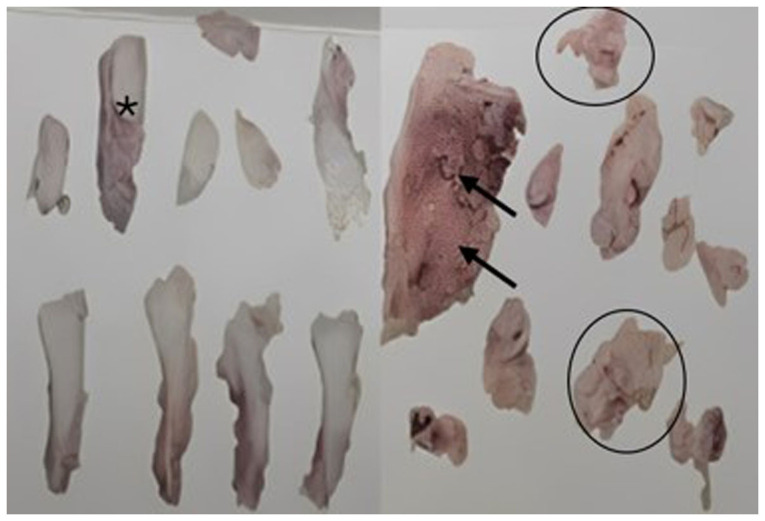
Anatomopathological study of the malleolus tumor of the right fibula. Data suggestive of osteosarcoma are observed in the bone (black arrows), periosteum (asterisk) and fibrofatty tissue (black circle). With abundant osteoid, fibrous stroma and fusiform cell conglomerates with one or more prominent nuclei, giant tumor cells and two mitoses are seen in 10 fields.

Subsequently, following appropriate preparation, the patient underwent a biopsy of the affected area. The excised tissue was sent for analysis. The histopathology report described fragmented tissue measuring 6x4.2x1.2 cm in total, with a white or pink color, rough or granular texture, soft or semi-solid consistency, and exhibiting portions of white and hard bone. Microscopically, features consistent with a malignant mesenchymal neoplasm affecting bone, periosteum and adjacent fibroadipose tissue were observed. Abundant osteoid and fibrosis were present. Clusters or fascicles of spindle, oval, irregular, or polygonal cells with nuclear enlargement, oval, elongated, or irregular nuclei, vesicular or hyperchromatic, with one or two prominent nucleoli, two mitoses in 10 high-power fields, and scant cytoplasm were observed in the osteoid and fibrous stroma. Multinucleated giant tumor cells were also noted. The diagnosis of OS involving bone, periosteum and neighboring fibroadipose tissue, fragmented, measuring 6x4.2x1.2 cm, was established (Fig. 4). Upon the confirmation of diagnosis, the patient was closely monitored. At approximately the 15th week of gestation, she was admitted to the Department of Traumatology and Orthopedics of Hospital Star Médica Chihuahua (Chihuahua, Mexico) for the resection of the distal third of the right fibula. Following the surgical protocol, anesthesia was induced by regional blockade, and hemostatic control was established. The patient was positioned in dorsal decubitus, and asepsis and antisepsis techniques were employed for the lower right limb. Sterile fields were prepared, and an approach was made to the lateral malleolus. The skin, subcutaneous tissue and fascia were dissected, and the area between the long and short fibula and the distal third of the fibula was approached. A granular-looking tumor measuring ~8x5 cm was located and excised. Post-surgical cleansing and conventional wound closure were performed without complications (Fig. 1B). Following recovery, after 1 week of her admission the patient exhibited good progress and was admitted for a scheduled outpatient follow-up. Analgesic management and antibiotic therapy with clindamycin at 300 mg every 6 h for 7 days were prescribed, along with general care of the surgical site and follow-up at the outpatient clinic. The patient subsequently continued with an uncomplicated prenatal follow-up. Between 29 and 31 weeks of gestation, adjuvant chemotherapy was initiated with doxorubicin at 110 mg in 100 cc of 0.9% saline solution and cisplatin at 140 mg in 1,000 cc of 0.9% saline solution. Additionally, anti-emetic, anti-inflammatory and anti-edematous management was provided, along with the administration of 6 mg pegfilgrastim following chemotherapy to prevent neutropenia.

**Figure 5:**
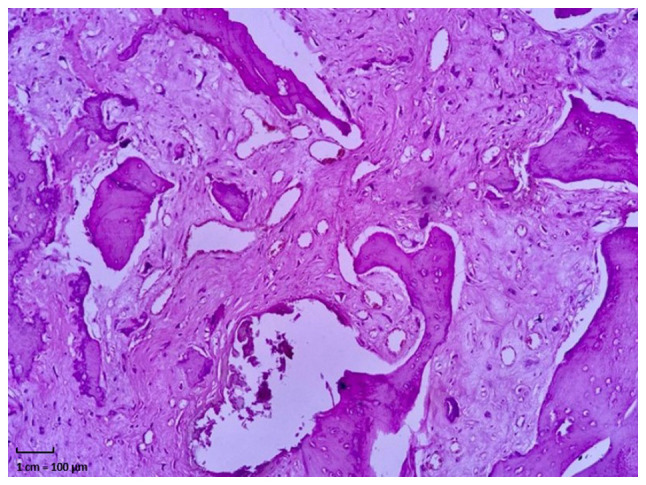
Anatomopathological study resulting from surgical resection. Diffuse parenchymal fibrosis is observed, without residual malignant neoplastic tissue. Scale bar, 1 cm (100 µm).

Following the completion of the chemotherapeutic regimen, the patient exhibited favorable progress and underwent a scheduled cesarean section at the beginning of the 37th week of gestation. The surgery was performed without complications, yielding a live product with a weight of 2,285 grams, a height of 48 cm, and Apgar scores of 9 at 1 and 5 min. At 1 week postpartum, the patient underwent three additional sessions of the same chemotherapeutic regimen. Subsequently, a biopsy was performed to evaluate the antitumor efficacy. A histopathological analysis revealed multiple bone fragments totaling 8x7 cm, characterized by a gray-brown, hard, irregular, opaque and trabecular appearance. The obtained sample was processed using 10% buffered formalin, then embedded in paraffin and sectioned into 50-µm-thick slices using a microtome. The sections were placed on slides and stained with hematoxylin and eosin; these procedures were performed in the Cytopathology and Oncological Pathology Laboratory, Histopath^®^, in accordance with standard guidelines. A microscopic examination revealed sections of cortical and medullary bone tissue with distinct intraparenchymal hemorrhage and diffuse fibrosis, alongside the loss of elements from all three hematopoietic series (myeloid, erythroid and platelets). Notably, no residual malignant neoplastic tissue was detected, indicating a complete pathological response (residual tumor, 0%) (Fig. 5).

A regards the follow-up of the gestational product, pediatric reports indicate a generally positive outcome, with only a few unrelated conditions. Notably, a cardiac murmur was diagnosed at 1 month of age, but was deemed benign. Upon the writing of the present manuscript, the patient has been asymptomatic for >1 year. Additionally, the infant experienced symptoms of gastroesophageal reflux, which led to nutritional issues, resulting in iron-deficiency anemia and proctocolitis with limited weight gain around the 5th month. All these conditions have been effectively treated. Currently, all symptoms have resolved, and there are no pathological findings linking the condition to in utero exposure to the antineoplastic drug or the maternal OS.

## Discussion

The present study describes the case of a young pregnant woman diagnosed with OS in the distal fibula, who was successfully treated with surgery and adjuvant chemotherapy. It is important to highlight that the woman in the present case report had no evident risk factors for developing OS. Thus, this clearly demonstrates the unpredictable nature of this neoplasm in young individuals. Fortunately, long-term follow-up reports for this woman indicate that both she and the gestational product are in good health, with no pathologies linked to the primary tumor or arising from the treatment. Notably, the occurrence of neoplasms during pregnancy is rare, with an incidence of ~1 case per 1,000 pregnancies (11). A previous review suggested that the incidence of OS in pregnant women may be on the rise due to the increasing trend of delaying pregnancy until later ages (12). However, the age of the patient in the present study does not align with this trend. Therefore, it can be hypothesized that the patient in the present case report likely had more subtle risk factors that predisposed her to develop this neoplastic lesion, probably of a polygenic nature or related to specific age-related risk factors. As regards the latter, a previous retrospective study observed that the age group of 15 to 25 years was the one with the most significant increase in OS diagnoses over recent decades compared with other age groups (13). Additionally, previous reports have documented incidental diagnoses of OS in pregnant women and women on reproductive age while they were being treated for other conditions (14,15). In the patient described herein, the clinical manifestations observed were consistent with the typical presentations of OS in the appendicular skeleton (16). The initial evaluation of the edema and pain involved an ankle X-ray, which revealed radiodense and lytic lesions typical of this neoplasm (17). Although positron emission tomography is the ideal diagnostic tool for identifying metastases (18), herein, its use was restricted due to the pregnancy of the patient. Consequently, contrast-enhanced MRI was employed, as it effectively visualizes the extent of soft tissue involvement and the overall disease progression (17,19). In the absence of metastasis, surgical treatment combined with adjuvant chemotherapy was selected. Previous studies have demonstrated that this approach can preserve the affected limb and ensure a favorable long-term prognosis (9,20). Although surgery is a valid approach for treating localized stages of osteosarcoma, it can cause localized cellular stress, potentially leading to immune dysregulation and increasing the risk of tumor recurrence over time (21). Considering this and the young age of the patient in the present case report, the medical team decided on a combined treatment plan of surgery and adjuvant chemotherapy. Drug management during pregnancy has historically been contentious; advancements in pharmacokinetics and pharmacovigilance now support the safe use of medications during pregnancy for both the mother and fetus (22,23). In addition, recent advancements in therapies, particularly those utilizing nanoparticles, have demonstrated their effectiveness in the treatment of OS (24). However, the availability of antineoplastic drugs in the authors' hospital (Hospital Star Médica Chihuahua) is limited. Consequently, the selection of the medication was based on the established therapeutic efficacy of the drug for this type of cancer. Since the 1970s, the use of cisplatin has been shown to be associated with improved survival rates in OS by 90% and extended relapse-free periods by 60% (25). However, in the case described herein, cisplatin and doxorubicin were selected, as it remains effective at low doses for these lesions (26-28). The follow-up data confirmed a satisfactory antitumor response in the patient, along with the birth of the product without significant complications. As regards the treatment used, while the management of OS depends on the tumor stage, it is also closely aligned with the treatment protocols specific to each hospital (29). Cytotoxic therapies used in OS include alkylating agents (ifosfamide and cisplatin), antimetabolites (methotrexate and gemcitabine), topoisomerase inhibitors (etoposide), anthracyclines (doxorubicin), or microtubule inhibitors. Additionally, recent studies suggest that PD-1 inhibitors may serve as an effective adjuvant immunotherapy for patients with OS who have already undergone standard chemotherapy; this treatment has the potential to increase survival rates and lower the risk of long-term tumor recurrence (30,31). Other potential treatments include RNA silencers and certain nucleotide analogs, which have proven effective in other types of solid tumors. However, these are still in phase I and II clinical trials: (NCT02985125, https://clinicaltrials.gov/study/NCT02985125; and NCT02981342, https://clinicaltrials.gov/study/NCT02981342, respectively) and will require time before they become standard practice (32). In the authors' opinion, the treatment selected for the patient in the present case report was the most suitable, considering her personal circumstances and the available resources at the hospital.

The present study has a notable limitation which should be mentioned: The routine diagnosis of OS typically relies on the immunohistochemical analysis of the tumor tissue. Commonly used molecular markers include vimentin, S100 protein and CD99, which collectively assist in determining the cellular origin and genetic lineage of the tumor. However, due to resource limitations at Hospital Star Médica Chihuahua, the diagnosis of OS was based on clinical presentation and histopathological findings from the tumor sample. While this diagnostic approach is generally deemed sufficient (33), the absence of specific markers hampers a comprehensive characterization of the neoplasm. For purposes of comparison, a summary of previously reported similar cases reports in the medical literature is provided in Table I (11,34-48). Future research is thus required to explore the utility of these markers as part of a thorough diagnostic screening for similar lesions. However, given the positive response of the patient to chemotherapy and the outcomes reported during the resection, it is expected that she will sustain a favorable long-term prognosis.

## Figures and Tables

**Table I:** Summary of previously reported similar cases reports in the medical literature.

Authors, year of publication	Characteristics	Site	Treatment	Outcomes	(Refs.)
Pratt *et al*, 1977	4 Women, unknown gestational age	No data	Surgical resection and chemotherapy: Adriamycin plus cyclophosphamide, following by high dose of methotrexate and leucovorin	50% rate survival, 1 patient without metastasis at follow-up	(34)
Haerr and Pratt, 1985	Young woman at 25th gestational week	Hip	Multiple chemotherapeutics regime	Newborn and mother survival at 4 years follow-up	(35)
Huvos *et al*, 1985	Young woman	Hip	No data	No data	(36)
Adair *et al*, 2001	Young woman at 16th gestational week	Bowel	No data	No data	(37)
Nakajima *et al*, 2004	Young woman at 25th gestational week	Long bones	Doxorubicin and ifosfamide	Newborn and mother survival at 8 months follow-up	(38)
Nepal *et al*, 2005	Young woman	No data	No data	No data	(39)
Koçak *et al*, (2006)	Young woman at 7th month of gestation	Heart	Mitral valve replacement	Newborn and mother survival at 3 months follow-up	(40)
Kanazawa *et al*, 2009	Women with acromegaly at 6th gestational week	Jaw	Surgical resection	No data	(41)
Han *et al*, 2011	Woman at second trimester of gestation	Femur	No data	Newborn and mother survival	(42)
Gonin *et al*, 2011	Woman at 26th gestational week	Adrenal gland	No data	No data	(43)
Corrêa *et al*, 2012	Young woman	No data	No data	No data	(44)
Ding *et al*, 2016	Young woman	Kidney	Nephrectomy and ‘conventional chemotherapy’	Dead by metastatic disease	(45)
Narla *et al*, 2018	Young woman	Breast	No data	No data	(46)
Figueiro-Filho *et al*, 2018	Ten case reports: Two during the pregnancy	Pelvis	No data	No data	(11)
Breda *et al*, 2021	Young woman	Breastbone	No data	No data	(47)
Regmi *et al*, 2023	Middle-aged woman at 1st month of pregnancy	Lung	No data	No data	(48)

## Data Availability

The datasets used and/or analyzed during the current study are available from the corresponding author on reasonable request.
